# Gastroscope-assisted endoscopic retrograde cholangiopancreatography with duodenal retroversion for management of recurrent biliary stent obstruction in malignant duodenal stricture

**DOI:** 10.1055/a-2836-1392

**Published:** 2026-03-26

**Authors:** Gianluca Franchellucci, Alessandro De Marco, Giacomo Marcozzi, Marco Spadaccini, Matteo Colombo, Alessandro Fugazza, Alessandro Repici

**Affiliations:** 1 9268 Department of Gastroenterology, IRCCS Humanitas Research Hospital, Rozzano, Milan, Italy; 2 18505 Endoscopy Unit, Humanitas Gavazzeni, Bergamo, Italy; 3 150525 Department of Biomedical Sciences, Humanitas University, Pieve Emanuele, Milan, Italy

The management of biliary obstruction in patients with an “endoscopically excluded” papilla resulting from malignant duodenal strictures or altered surgical anatomy presents a significant clinical challenge. Several therapeutic strategies can be employed to achieve biliary decompression in these complex scenarios 1 .

Endoscopic ultrasound (EUS)-guided biliary drainage, such as EUS-guided hepaticogastrostomy (EUS-HGS), is increasingly considered a first-line alternative to transpapillary access. This approach is associated with lower rates of adverse events compared to percutaneous transhepatic biliary drainage 2 3 . Other viable options include the placement of a duodenal stent to restore luminal patency or the use of EUS-guided gastroenterostomy to create a bypass for subsequent entero-endoscopic retrograde cholangiopancreatography (ERCP). Furthermore, ERCP performed with a thin, forward-viewing endoscope can be utilized to traverse or bypass duodenal stenoses.

Although EUS-guided techniques are increasingly favored, they are technically complex, should be performed only by expert operators, and require an adequate acoustic window. When such techniques are not feasible or in cases of complex anatomy, gastroscope-assisted ERCP remains an effective salvage strategy, as demonstrated in the case reported here. The literature suggests that, in carefully selected patients, a gastroscope can achieve success rates comparable to those of a duodenoscope. However, it presents inherent technical limitations, including the absence of elevator and increased difficulty in performing standard sphincterotomy 4 5 .

We report the case of a 54-year-old patient diagnosed with locally advanced adenocarcinoma of the pancreatic head, treated in April 2025 with ERCP and placement of a 10 × 60 mm partially covered metal biliary stent. Three months later, the patient developed symptoms of gastric outlet obstruction due to duodenal bulb stenosis caused by tumoral infiltration, and a GE was performed.

Approximately 1 month later, the patient presented with fever, jaundice and abdominal pain. Abdominal computed tomography showed dilation of the intrahepatic bile ducts with signs of obstruction of the previously placed biliary stent. An attempt to perform EUS-HGS was therefore made. During the procedure, after multiple unsuccessful attempts to access the left intrahepatic bile duct due to the lack of an adequate EUS window, the strategy was changed. An entero-ERCP approach through the GE was attempted but was unsuccessful due to the narrow angulation of the jejunal loop. As traversal of the duodenal bulb stenosis with a duodenoscope was not possible, a gastroscope with an external diameter of 7.9 mm and a 3.2-mm working channel (Fujifilm EG-840TP, Japan) was used to reach the papillary area, avoiding the need for stenosis dilation. The distal end of the previously placed metal biliary stent was identified and appeared obstructed by tumoral ingrowth ( Video 1 ). Standard cannulation using a sphincterotome and a guidewire was attempted but was unsuccessful because of inadequate angulation. Taking advantage of the endoscope’s excellent maneuverability and flexibility, a retroversion maneuver in the second portion of the duodenum was performed, allowing the successful cannulation of the main bile duct through the stent. Cholangiography confirmed tumoral ingrowth in the mid-distal segment of the stent and a partially covered metal biliary stent (10 × 80 mm) was placed using a stent-in-stent technique. Technical success was confirmed by marked aerobilia in the intrahepatic bile ducts. No immediate adverse events occurred. Following the procedure, the patient experienced a rapid resolution of jaundice and clinical symptoms. At a short-term follow-up, biliary drainage remained effective, significantly improving the patient’s quality of life during palliative care.

Utilizing a thin, forward-viewing endoscope allows access to the papilla and biliary cannulation, successfully bypassing duodenal obstruction. The retroversion maneuver facilitates biliary cannulation through the obstructed stent and the placement of a new stent-in-stent.Video 1

This video demonstrates that, in cases of duodenal obstruction and failure of advanced EUS-guided biliary drainage techniques, facilitated access to the papilla and biliary cannulation can be achieved using thin, forward-viewing endoscopes. Retroversion in the second portion of the duodenum may help overcome technical limitations, such as the lack of an elevator, by allowing alignment of the endoscope along the bile duct axis.

Endoscopy_UCTN_Code_TTT_1AR_2AB Endoscopy_UCTN_Code_TTT_1AS_2A
